# Trace proteinuria as a risk factor for cancer death in a general population

**DOI:** 10.1038/s41598-021-96388-3

**Published:** 2021-08-19

**Authors:** Masaru Matsui, Kazuhiko Tsuruya, Hisako Yoshida, Kunitoshi Iseki, Shouichi Fujimoto, Tsuneo Konta, Toshiki Moriyama, Kunihiro Yamagata, Ichiei Narita, Masato Kasahara, Yugo Shibagaki, Masahide Kondo, Koichi Asahi, Tsuyoshi Watanabe

**Affiliations:** 1 Department of Nephrology, Nara Medical University, Kashihara, Nara, Japan; 2 Department of Nephrology, Nara Prefecture General Medical Center, 2-897-5 Shichijo-nishimachi, Nara, Japan; 3 Steering Committee of Research on Design of the Comprehensive Health Care System for Chronic Kidney Disease (CKD), Based on the Individual Risk Assessment by Specific Health Checkup, The Ministry of Health, Labour and Welfare of Japan, Fukushima, Japan; 4 Department of Medical Statistics, Osaka City University Graduate School of Medicine, Osaka, Japan

**Keywords:** Kidney diseases, Cancer, Nephrology

## Abstract

Growing evidence has demonstrated an association between nondialysis chronic kidney disease and cancer incidence, although the association between trace proteinuria and cancer death remains unclear. The aim of this study was to investigate the association between trace proteinuria and cancer death in a community-based population in Japan. This was a prospective cohort study of 377,202 adults who participated in the Japanese Specific Health Check and Guidance System from 2008 to 2011. Exposure was dipstick proteinuria categorized as − (negative), ± (trace), 1 + (mild), or ≥ 2 + (moderate to heavy). Outcome was cancer death based on information from the national database of death certificates. Adjusted Cox hazard regression model was used to evaluate the associations between trace proteinuria and cancer death. During median follow-up of 3.7 years, 3056 cancer deaths occurred, corresponding to overall cancer death rate of 21.7/10,000 person-years. In the fully adjusted model, risk of cancer death increased significantly in each successive category of proteinuria: hazard ratio (HR) (95% confidence interval [95% CI]) for risk of cancer death was 1.16 (1.03–1.31), 1.47 (1.27–1.70), and 1.61 (1.33–1.96) for trace, mild, and moderate to heavy proteinuria, respectively. Sensitivity analyses revealed a similar association between trace proteinuria and cancer death, and participants with trace proteinuria had greater risk of mortality from hematological cancers (HR: 1.59 [95% CI: 1.09–2.31]). Both mild to heavy and trace proteinuria were significantly associated with risk of mortality from cancer in a general population.

## Introduction

Numerous complications from chronic kidney disease (CKD) are recognized worldwide as being associated with loss of quality of life and poor prognosis. Cardiorenal syndrome is an established CKD-associated condition whereby a complex interrelationship between cardiac and renal dysfunction amplifies the progression of impairment and failure of both organs. Associations have emerged between increasing severity of CKD and other conditions, such as infection, anemia, and mineral bone disorder. Additionally, growing evidence from a population-based longitudinal study suggests nondialysis CKD patients have a high risk of cancer incidence^1,2^.

A recent observational study found a J-shaped relationship between estimated glomerular filtration rate (eGFR) and incidence of any cancer, with lowest risk at 45–59 mL/min/1.73 m^2^^3^. A larger retrospective cohort study showed that reduced eGFR is independently associated with renal and urothelial cancer but not with other cancer types^4^. In contrast, a few reports demonstrated associations of albuminuria or proteinuria with cancer incidence and mortality^3,5–7^. Two studies from Korea revealed subjects with high proteinuria over 1 + using dipstick test have an approximately 1.1-fold greater risk of cancer incidence^3,6^. Based on other two observational studies, urinary albumin to creatinine ratio was also associated with increased cancer incidence, with a dose–response relationship^5,7^. There has been only one report in Korea, showing a strong relation between proteinuria and cancer mortality^8^, although it remains incompletely understood.

In the general population, the clinical utility of trace proteinuria for screening microalbuminuria and predicting all-cause and cardiovascular mortality has been established ^9,10^. However, in earlier studies on associations between proteinuria and cancer risks, subjects with negative or trace proteinuria were analyzed in combination, and were not considered to have proteinuria. Consequently, the effects of trace proteinuria on adverse events were not described^3,11^. We therefore conducted a prospective observational study using a nationwide database, to examine the association between trace proteinuria and cancer-specific death in a community-based population in Japan.

## Results

### Proteinuria and overall cancer death

A total of 377,202 subjects met the criteria for eligibility for analysis. Median age (interquartile range) of participants was 64 (58–69) years, and 167,006 (44%) were men. Overall, 32,051 (9%) and 22,765 (7%) participants had trace and overt proteinuria, respectively, including 29,774 (8%) with eGFR < 60 mL/min/1.73 m^2^. Baseline parameters according to proteinuria level are shown in Table 1.

**Table 1:** Baseline parameters according to level of proteinuria.

	Proteinuria			
	Negative ( −)	Trace ( ±)	Mild (1 +)	Moderate to Heavy (≥ 2 +)
No. of participants	322,386	32,051	15,297	7468
Age, years	64 (58–69)	64 (56–69)	65 (58–70)	65 (60–70)
Sex (male), %	42.2	52.7	59.4	66.2
Body mass index, kg/m^2^	23.1 (21.0–25.4)	23.8 (21.6–26.3)	24.6 (22.1–27.1)	25.1 (22.7–27.8)
Current smoker, %	15.2	19.5	21.8	22.9
Alcohol habit, %				
Non-drinker	23.1	26.4	28.1	27.3
Occasional-drinker	22.5	24.5	23.3	22.6
Daily drinker	54.4	49.2	48.6	50.1
Systolic BP, mmHg	128 (118–139)	130 (120–140)	134 (123–146)	139 (128–150)
Diastolic BP, mmHg	76 (70–82)	78 (70–84)	80 (70–87)	80 (72–89)
eGFR, mL/min/1.73 m^2^	89 (75–106)	86 (73–104)	83 (65–101)	73 (53–89)
HbA1c, %	5.3 (5.0–5.5)	5.3 (5.0–5.7)	5.4 (5.1–5.9)	5.5 (5.1–6.4)
LDL-cholesterol, mg/dL	123 (104–144)	122 (102–144)	122 (101–14)	122 (101–144)
AST, U/L	22 (19–27)	23 (19–28)	23 (19–30)	23 (19–30)
Previous cardiovascular diseases	5.0	5.9	8.0	10.2
Antihypertensive drugs. %	29.6	36.9	47.9	59.6

Data are presented as median (interquartile range) or n (%). Abbreviation: AST, aspartate aminotransferase; BP, blood pressure; eGFR, estimated glomerular filtration rate; HbA1c, glycated hemoglobin; LDL, low-density lipoprotein.

During median follow-up of 3.7 years, 5979 deaths (including 3056 cancer deaths) occurred, corresponding to overall cancer death rate of 21.7/10,000 person-years. Cancer deaths occurred in 2456 of 322,386 (0.8%) participants with negative proteinuria (20.0 deaths/10,000 person-years), 289 of 32,051 (0.9%) with trace proteinuria (26.8 deaths/10,000 person-years), 198 of 15,297 (1.3%) with mild proteinuria (40.5 deaths/10,000 person-years), and 113 of 7468 (1.5%) with moderate to heavy proteinuria (48.7 deaths/10,000 person-years). This corresponded to unadjusted hazard ratio (HR) (95% confidence interval [95% CI]) of 1.30 (1.15–1.47), 1.89 (1.63–2.18), and 2.29 (1.90–2.77) in patients with trace, mild, and moderate to heavy proteinuria, respectively (Table 2).

**Table 2:** Hazard ratios for cancer death according to level of proteinuria.

	Proteinuria			
	Negative (−)	Trace (±)	Mild (1+)	Moderate to Heavy (≥ 2 +)
No. of deaths	2456	289	198	113
Cancer death rate, per 10,000 person-years	20.0	26.8	40.5	48.7
Unadjusted model	Reference	1.30 (1.15–1.47)	1.89 (1.63–2.18)	2.29 (1.90–2.77)
Model 1	Reference	1.20 (1.06–1.35)	1.57 (1.36–1.82)	1.75 (1.44–2.11)
Model 2	Reference	1.21 (1.07–1.37)	1.60 (1.38–1.85)	1.79 (1.48–2.16)
Model 3	Reference	1.16 (1.03–1.31)	1.47 (1.27–1.70)	1.61 (1.33–1.96)

Model 1 adjusted for age and sex. Model 2 adjusted for covariates in model 1 plus body mass index, current smoker, and alcohol consumption. Model 3 adjusted for covariates in model 2 plus systolic blood pressure, estimated glomerular filtration rate, hemoglobin A1c, low-density lipoprotein cholesterol, and aspartate aminotransferase.

In the fully adjusted model, risk of cancer death increased significantly in each successive category of proteinuria. HR (95% CI) for risk of cancer death was 1.16 (1.03–1.31), 1.47 (1.27–1.70), and 1.61 (1.33–1.96) for trace, mild, and moderate to heavy proteinuria, respectively.

### Subgroup and sensitivity analyses for overall cancer death

**Figure 1:**
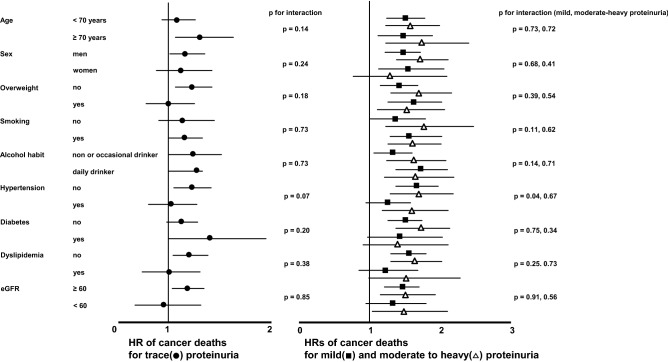
Multivariable-adjusted hazard ratio (95% confidence interval) of cancer death for trace, mild, and moderate to heavy versus negative proteinuria within subgroups stratified by age, sex, overweight, smoking, alcohol habit, hypertension, diabetes mellitus, dyslipidemia, and eGFR.

As shown in Fig. 1, the associations between mild and moderate to heavy proteinuria and risk of cancer death were similar in participants stratified by age, sex, overweight, smoking, alcohol habit, hypertension, diabetes mellitus, dyslipidemia, and eGFR. Participants with trace proteinuria tended to have greater risk of cancer death than those with negative proteinuria in subgroups with older individuals, males, current smokers, diabetes mellitus, and without overweight, hypertension, dyslipidemia, and eGFR. Results from sensitivity analyses were also similar to those from our main analysis when we restricted the analysis to participants followed-up for at least 3 months after enrollment (HR: 1.16 [95% CI: 1.03–1.32]); when plasma levels of hemoglobin were included in the fully adjusted model (HR: 1.37 [95% CI: 1.13–1.67]); when history of cardiovascular events was included in the fully adjusted model (HR: 1.17 [95% CI: 1.03–1.32]); and when antihypertensive agents were included in the fully adjusted model (HR: 1.16 [95% CI: 1.03–1.32]).

### Trace proteinuria and site-specific cancer death

**Figure 2:**
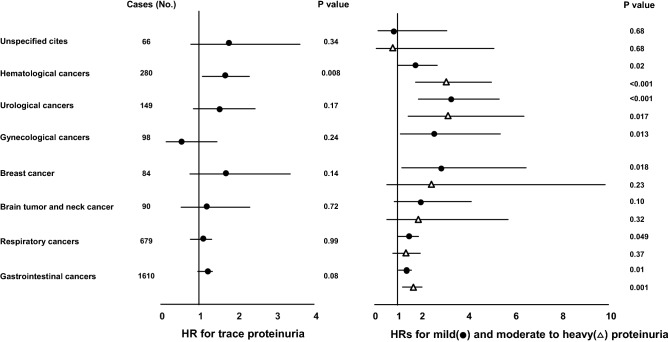
Multivariable-adjusted hazard ratio (95% confidence interval) of site-specific cancer death for trace, mild, and moderate to heavy versus negative proteinuria. * Data were not available due to the low number of gynecological cancer deaths in subjects with moderate to heavy proteinuria.

Mild and moderate to severe proteinuria demonstrated strong and similar associations with increased risk of hematological, urological, and gastrointestinal cancers (Fig. 2). However, only hematological cancers reached significance (HR: 1.59 [95% CI: 1.09–2.31]) in participants with trace proteinuria. Stratified analysis by demographics for each site-specific cancer death was shown in Supplementary Table.

## Discussion

Herein, we emphasize a significant association between trace proteinuria and overall cancer death identified from data from a nationwide prospective cohort study. This association persisted following adjustment for lifestyle risk factors, hypertension, diabetes mellitus, dyslipidemia, and renal function. Similar results were obtained in sensitivity analyses, while analysis of mortality from site-specific cancers revealed a significant association between trace proteinuria and hematological cancers, but not other cancers. Mild to heavy proteinuria was associated with greater risk of cancer death than trace proteinuria, suggesting the risk of cancer death increased in each successive grade of dipstick-determined proteinuria.

According to an investigation conducted in Japan, subjects with dipstick-determined trace proteinuria had intermediate risk of experiencing hypertension, diabetes mellitus, and metabolic syndrome in any eGFR category. The authors suggested metabolic syndrome components should be investigated in subjects with trace proteinuria (even in those with normal eGFR) for cardiovascular risk reduction^12^. Studies from general populations participating in the Framingham Heart Study and Korean nationwide health screening program revealed trace proteinuria is a significant risk factor for all-cause mortality and cardiovascular mortality^13,14^. In the present study, compared with subjects with negative proteinuria, patients with trace proteinuria had a 1.16-fold greater risk of overall cancer death in models adjusted for clinical and demographic parameters, lifestyle habits, and laboratory findings.

Cancer is a major cause of mortality in developed countries, although predictors of overall cancer death are not fully established. Recently, the specialized area of nephrology known as onco-nephrology has emerged. It describes the complex links between cancer and the kidneys that have been identified as highly important for prognosis and quality of life in cancer patients. A Korean nationwide study of 242,583 participants showed that eGFR < 45 mL/min/1.73 m^2^ was significantly associated with kidney and ureteral cancers, multiple myeloma, and leukemia, whereas proteinuria ≥ 1 + was associated with a broader set of cancers^3^. Other studies also have revealed a strong relationship of proteinuria with the development of lung and urological cancers^5,7^ and death due to cancers of stomach, liver, pancreas, and lung, and myeloma^8^. In the study we found a relationship of proteinuria ≥ 1 + with mortality due to hematological, urological, gynecological, and gastrointestinal cancers. As shown in subgroup analyses stratified by demographics this relation seems to be stronger in hematological and urological caners. We also showed that participants with trace proteinuria had a greater risk of mortality from hematological cancers than other forms of cancer. In the clinical setting, minimal change nephrotic syndrome occurs as a paraneoplastic manifestation of classical Hodgkin’s disease, and urinary Bence-Jones protein is released from multiple myeloma. A firm link between hematological cancers and proteinuria may therefore exist.

We propose two hypotheses regarding direct and indirect mechanisms underlying the association between trace proteinuria and cancer death. First, the production or activation of cytokines such as angiogenic factors in the proinflammatory milieu associated with trace proteinuria may increase the risk of mortality from cancer via cancer cell invasiveness^15,16^. Second, trace proteinuria is both a marker of glomerular endothelial dysfunction and an early marker of systemic vascular endothelial dysfunction^17^. Therefore, accelerated angiogenesis and vasculogenesis triggered by vascular endothelial dysfunction may result in metastatic spread of cancer cells^18,19^. Further clinical research is required to investigate the association between trace proteinuria and cancer death.

This study has several limitations. First, because study participants were exclusively residents of Japanese prefectures, the results may not be generalizable to other populations. Second, the aim of this nationwide health check-up and guidance system (The Ministry of Health, Labour and Welfare; https://www.mhlw.go.jp/english/) is to detect metabolic syndrome and if confirmed, to provide individual instruction to modify lifestyle and the necessary treatment, which results in the reduction of cardiovascular diseases, stroke, and end-staging kidney diseases. Hence, the assessment regarding cancers including an interview on previous cancers or cancer at baseline were not addressed. we did not exclude participants with previous cancers or cancer at baseline, and patient backgrounds may have affected the results. Third, the diagnostic accuracy of dipstick urinalysis for proteinuria may lack reliability^20^, although dipstick-determined trace or negative proteinuria have high negative predictive value in the general population, with minimal risk of missed diagnosis of macroalbuminuria^21^. A recent meta-analysis has also demonstrated dipstick-determined protein can predict albuminuria and may help in CKD screening and prognosis^22^.

In conclusion, both mild to heavy and trace proteinuria were significantly associated with risk of mortality from cancer in a general population, suggesting screening for cancer may be necessary for early cancer intervention in subjects with trace proteinuria.

## Methods

### Study participants

The present investigation was part of a prospective study entitled ‘‘Study on the design of the comprehensive health care system for CKD based on the individual risk assessment by Specific Health Checkups,’’ and was based on data obtained from the Japanese Specific Health Check and Guidance System from 2008 to 2011 as previously described^23,24^. Beginning from 2008 in Japan, this ongoing annual health check program promotes early diagnosis and intervention strategies to prevent metabolic syndrome. This study included 664,926 subjects across seven prefectures who participated in the annual health check program between 2008 and 2014. We excluded subjects missing data on dipstick-determined proteinuria (N = 1745) or other baseline parameters (N = 285,979), resulting in a final study population of 377,202 participants. This study was conducted in accordance with the Private Information Protection Law and ethical guidelines for epidemiology research published by the Japanese Ministry of Health, Labour and Welfare in 2008.

### Data collection and clinical definitions

All participants completed a self-administered questionnaire that documented their medical history including previous cardiovascular diseases, current medications, smoking habit (current smoker or not), and alcohol consumption. Alcohol consumption was divided into three categories as previously described: nondrinker, occasional drinker, and daily drinker^25^. Blood pressure measurements and blood and urine sampling were performed at each participant’s local medical institute, as stipulated by the health check program. Blood samples were collected after participants had fasted overnight, and were analyzed using automated clinical chemical analyzers within 24 h of sampling.

Urinary protein excretion was examined by dipstick testing and categorized into four levels: − (negative), ± (trace), 1 + (mild), and ≥ 2 + (moderate to heavy). A Japanese equation was used to estimate the GFR: eGFR = 194 × (serum creatinine) − 1.094 × (age) − 0.287 (× 0.739 if female)^26^. Hypertension was defined as systolic blood pressure ≥ 140 mmHg and/or diastolic blood pressure ≥ 90 mmHg, or self-reported use of antihypertensive drugs. Diabetes mellitus was defined in accordance with the guidelines of the American Diabetes Association^27^: fasting glucose concentration ≥ 126 mg/dL, hemoglobin A1c level ≥ 6.5%, or self-reported use of antihyperglycemic drugs. Overweight was defined as body mass index ≥ 25 kg/m^2^. Dyslipidemia was defined as triglycerides ≥ 150 mg/dL, low-density lipoprotein cholesterol ≥ 140 mg/dL, high-density lipoprotein cholesterol < 40 mg/dL, or self-reported use of lipid-lowering drugs.

### Outcome

The primary endpoint of this observational study was time to cancer death. Date of death and causes of death between 2008 and 2014 were obtained from the national database of death certificates as previously described^28^.

### Statistical analysis

Baseline data are presented as median and interquartile range. A Cox hazard regression model was used to assess for unadjusted and adjusted associations between proteinuria and cancer death. Subjects with negative proteinuria were considered the reference group.

Candidate variables for adjustment were selected because of their close associations with cancer mortality^29–32^. We initially adjusted for patient demographic parameters, including age and sex, in model 1. Model 2 consisted of model 1 plus body mass index, current smoker, and alcohol consumption. Model 3 consisted of model 2 plus systolic blood pressure, eGFR, hemoglobin A1c, low-density lipoprotein cholesterol, and aspartate aminotransferase.

We examined whether the association between proteinuria and cancer death was similar in subgroups stratified by age, sex, overweight, smoking, alcohol habit, hypertension, diabetes mellitus, dyslipidemia, and eGFR. We further assessed the robustness of our results using sensitivity analyses. Multivariable-adjusted hazard analysis for cancer death was repeated with the following four sensitivity analyses. The first sensitivity analysis was restricted to participants with at least 3 months of follow-up. The remaining three analyses individually included plasma levels of hemoglobin, history of cardiovascular events, and antihypertensive agents in the fully adjusted model. Finally, we analyzed the relationship between proteinuria and site-specific cancer death.

A two-sided *P* < 0.05 was considered statistically significant. All statistical analyses were performed using IBM SPSS version 24.0 (IBM Corp., Armonk, NY, USA).

### Ethical approval and informed consent

The research protocol had been approved by the Ethics Committee of Fukushima Medical University (#1485 and #2771) and all procedures performed in studies involving human participants were also in accordance with its ethical standards and with 1964 Helsinki declaration and its amendments or comparable ethical standards. This study was conducted also in accordance with the Private Information Protection Law and ethical guidelines for epidemiology research published by the Japanese Ministry of Health, Labour and Welfare in 2008 [http://www.mhlw.go.jp/file/06-Seisakujouhou-10600000-Daijinkanboukouseikagakuka/0000069410.pdf]. Informed Consent was waived by the Ethics Committee of Fukushima Medical University. Instead, we publicized information concerning this study on the web [http://www.fmu.ac.jp/univ/sangaku/data/koukai_2/2771.pdf] and ensured the opportunities for the research subjects to refuse utilizing their personal information.

## Supplementary Information

Supplementary Information.
