# Supplementary material for: Trace proteinuria as a risk factor for cancer death in a general population
Source: Sci Rep. 2021 Aug 19;11:16890. doi: 10.1038/s41598-021-96388-3 (PMC8376860; doi:10.1038/s41598-021-96388-3)
Supplement: Supplementary file 1 — Supplementary Information. [file 41598_2021_96388_MOESM1_ESM.docx]

**Supplementary Table 1: Fully adjusted HR of each cancer death for trace, mild and moderate-heavy versus negative proteinuria in subgroup analyses.**

**Hematological Cancers**

| Demographics | | trace proteinuria | | mild proteinuria | | moderate to heavy proteinuria | |
| --- | --- | --- | --- | --- | --- | --- | --- |
|  | | HRs(95%CIs) | p | HRs(95%CIs) | p | HRs(95%CIs) | p |
| age | < 70yrs | 1.25(0.76-2.07) | 0.38 | 1.76(0.97-3.20) | 0.06 | **2.58(1.27-5.21)** | **0.009** |
|  | ≥ 70yrs | **2.33(1.32-4.12)** | **0.004** | 1.47(0.62-3.46) | 0.38 | **3.53(1.59-7.85)** | **0.002** |
| sex | male | 1.48(0.92-2.38) | 0.11 | 1.75(0.62-3.46) | 0.38 | **2.67(1.41-5.05)** | **0.003** |
|  | female | 1.79(0.98-3.28) | 0.06 | 1.29(0.47-3.54) | 0.63 | **3.82(1.50-9.72)** | **0.005** |
| hypertension | no | **1.87(1.24-2.83)** | **0.003** | **1.92(1.10-3.35)** | **0.02** | **2.06(0.94-4.50)** | **0.02** |
|  | yes | 0.85(0.34-2.15) | 0.74 | 1.06(0.38-2.96) | 0.92 | **4.69(2.27-9.68)** | **<0.001** |
| diabetes | no | **1.56(1.05-2.31)** | **0.03** | 1.57(0.92-2.67) | 0.10 | **2.83(1.58-5.07)** | **<0.001** |
|  | yes | 2.04(0.54-7.67) | 0.29 | 2.42(0.62-9.52) | 0.20 | 2.98(0.76-11.6) | 0.12 |
| eGFR | ≥ 60ml/min/1.73m^2^ | **1.57(1.04-2.36)** | **0.03** | 1.50(0.83-2.71) | 0.18 | **2.27(1.05-4.90)** | **0.04** |
|  | < 60ml/min/1.73m^2^ | 1.61(0.65-3.97) | 0.30 | 1.84(0.71-4.74) | 0.21 | **5.30(2.26-12.4)** | **<0.001** |

**Urological Cancers**

| Demographics | | trace proteinuria | | mild proteinuria | | moderate to heavy proteinuria | |
| --- | --- | --- | --- | --- | --- | --- | --- |
|  | | HRs(95%CIs) | p | HRs(95%CIs) | p | HRs(95%CIs) | p |
| age | < 70yrs | 1.52(0.82-2.79) | 0.18 | **2.36(1.17-4.75)** | **0.02** | **3.83(1.73-8.56)** | **0.001** |
|  | ≥ 70yrs | 1.25(0.44-3.56) | 0.68 | **4.98(2.20-11.3)** | **<0.001** | 1.05(0.13-8.34) | 0.97 |
| sex | male | 1.18(0.65-2.15) | 0.59 | **2.59(1.44-4.66)** | **0.002** | **3.25(1.54-6.85)** | **0.002** |
|  | female | **4.12(1.35-12.6)** | **0.01** | **10.1(3.21-31.9)** | **<0.001** | N/A |  |
| hypertension | no | 1.61(0.87-2.97) | 0.13 | **4.47(2.54-7.86)** | **<0.001** | **3.40(1.33-8.67)** | **0.01** |
|  | yes | 1.12(0.40-3.19) | 0.83 | 0.96(0.23-4.07) | 0.96 | 2.78(0.83-9.37) | 0.10 |
| diabetes | no | 1.06(0.57-1.98) | 0.85 | **3.19(1.87-5.45)** | **<0.001** | **2.99(1.37-6.54)** | **0.006** |
|  | yes | **10.8(2.53-46.2)** | **0.001** | 3.75(0.35-40.0) | 0.27 | 6.27(0.44-89.9) | 0.18 |
| eGFR | ≥ 60ml/min/1.73m^2^ | 1.18(0.63-2.21) | 0.61 | **2.63(1.39-4.95)** | **0.003** | 1.26(0.31-5.19) | 0.75 |
|  | < 60ml/min/1.73m^2^ | **2.98(1.06-8.42)** | **0.04** | **5.31(1.92-14.7)** | **0.001** | **9.49(3.09-29.1)** | **<0.001** |

**Respiratory Cancers**

| Demographics | | trace proteinuria | | mild proteinuria | | moderate to heavy proteinuria | |
| --- | --- | --- | --- | --- | --- | --- | --- |
|  | | HRs(95%CIs) | p | HRs(95%CIs) | p | HRs(95%CIs) | p |
| age | < 70yrs | 0.75(0.52-1.08) | 0.12 | 1.27(0.86-1.89) | 0.23 | 1.33(0.78-2.27) | 0.29 |
|  | ≥ 70yrs | **1.67(1.11-2.50)** | **0.01** | 1.62(0.96-2.74) | 0.07 | 1.08(0.46-2.16) | 0.86 |
| sex | male | 1.04(0.77-1.40) | 0.81 | 1.44(1.03-2.02) | 0.04 | 1.24(0.77-2.00) | 0.38 |
|  | female | 0.92(0.50-1.69) | 0.79 | 1.01(0.41-2.46) | 0.99 | 1.03(0.25-4.20) | 0.97 |
| hypertension | no | 0.99(0.71-1.39) | 0.96 | 1.45(0.97-2.17) | 0.07 | 1.49(0.83-2.68) | 0.18 |
|  | yes | 1.06(0.67-1.68) | 0.79 | 1.26(0.76-2.11) | 0.37 | 0.99(0.49-2.00) | 0.98 |
| diabetes | no | 0.96(0.72-1.29) | 0.80 | 1.37(0.97-1.94) | 0.07 | 1.17(0.68-2.00) | 0.57 |
|  | yes | 1.34(0.67-2.70) | 0.41 | 1.37(0.63-2.99) | 0.43 | 1.58(0.68-3.71) | 0.29 |
| eGFR | ≥ 60ml/min/1.73m^2^ | 1.04(0.77-1.39) | 0.80 | **1.45(1.02-2.06)** | **0.04** | 1.25(0.70-2.24) | 0.45 |
|  | < 60ml/min/1.73m^2^ | 0.85(0.42-1.71) | 0.65 | 0.91(0.44-1.88) | 0.80 | 0.94(0.42-2.09) | 0.88 |

**Gastrointestinal Cancers**

| Demographics | | trace proteinuria | | mild proteinuria | | moderate to heavy proteinuria | |
| --- | --- | --- | --- | --- | --- | --- | --- |
|  | | HRs(95%CIs) | p | HRs(95%CIs) | p | HRs(95%CIs) | p |
| age | < 70yrs | 1.17(0.96-1.43) | 0.11 | **1.29(1.00-1.67)** | **0.049** | 1.36(0.97-1.90) | 0.08 |
|  | ≥ 70yrs | 1.09(0.79-1.56) | 0.59 | 1.28(0.87-1.87) | 0.21 | **2.04(1.32-3.13)** | **0.001** |
| sex | male | 1.19(0.98-1.45) | 0.07 | **1.34(1.06-1.20)** | **0.01** | **1.73(1.30-2.30)** | **<0.001** |
|  | female | 1.02(0.72-1.44) | 0.92 | 1.07(0.66-1.72) | 0.67 | 0.88(0.39-1.99) | 0.76 |
| hypertension | no | 1.21(0.99-1.48) | 0.06 | **1.32(1.01-1.73)** | **0.04** | 1.70(1.19-2.42) | 0.053 |
|  | yes | 1.02(0.75-1.38) | 0.93 | 1.25(0.88-1.76) | 0.21 | 1.43(0.96-2.14) | 0.38 |
| diabetes | no | 1.13(0.94-1.35) | 0.20 | 1.27(0.99-1.61) | 0.05 | 1.79(1.33-2.41) | <0.001 |
|  | yes | 1.24(0.80-1.92) | 0.33 | 1.30(0.40-1.10) | 0.11 | 1.01(0.56-1.82) | 0.97 |
| eGFR | ≥ 60ml/min/1.73m^2^ | **1.21(1.01-1.44)** | **0.04** | 1.23(0.96-1.56) | 0.10 | **1.55(1.16-2.17)** | **0.01** |
|  | < 60ml/min/1.73m^2^ | 0.75(0.46-1.23) | 0.25 | 1.21(0.78-1.89) | 0.39 | 1.07(0.65-1.77) | 0.78 |
